# Cost‐effectiveness and patient‐reported outcomes of combined versus isolated robotic rectal and pelvic organ prolapse repair: A multicentre prospective cohort study

**DOI:** 10.1111/codi.70570

**Published:** 2026-08-02

**Authors:** Shannon L. Wallace, James W. Ogilvie, Liliana Bordeianou, Michelle Earley, Raisa Platte, Milena M. Weinstein, Eric R. Sokol, Ekene A. Enemchukwu, Kavita Mishra, Brooke H. Gurland

**Affiliations:** ^1^ Division of Urogynaecology and Pelvic Floor Disorders, Department of Obstetrics and Gynaecology Cleveland Clinic Foundation Cleveland Ohio USA; ^2^ Division of Colorectal Surgery and Pelvic Floor Reconstruction Corewell Health Grand Rapids Michigan USA; ^3^ Department of Surgery, Pelvic Floor Disorders Center Massachusetts General Hospital and Harvard Medical School Boston Massachusetts USA; ^4^ Department of Surgery, Pelvic Health Center Stanford University Stanford California USA; ^5^ Department of Gynecology Stanford University Stanford California USA; ^6^ Department of Urology, Pelvic Health Center Stanford University Stanford California USA

**Keywords:** cost‐effectiveness analysis, patient‐reported outcome measures, pelvic organ prolapse, rectal prolapse, robotic surgical procedures, ventral mesh rectopexy

## Abstract

**Aim:**

Rectal prolapse (RP) and pelvic organ prolapse (POP) frequently coexist, yet are often evaluated and treated separately. This study aimed to compare patient‐reported outcomes and cost‐effectiveness of combined versus isolated robotic prolapse repair.

**Methods:**

We conducted a prospective multicentre cohort study across three tertiary pelvic floor centres. Women undergoing robotic rectal prolapse repair (rRP), robotic pelvic organ prolapse repair (rPOP) or combined robotic repair (rRP + rPOP) were included. Validated patient‐reported outcome measures were collected at baseline and 12 months. Health utility was derived from EQ‐5D‐3L and used to calculate quality‐adjusted life years (QALYs). A trial‐based cost‐effectiveness analysis was performed from the healthcare sector perspective. A theoretical cohort was modelled to examine staged versus combined repair in patients with multicompartment prolapse.

**Results:**

Forty‐seven patients were included (rRP *n* = 14, rPOP *n* = 16, rRP + rPOP *n* = 17). All groups demonstrated significant improvement in pelvic floor symptoms and quality of life at 12 months. Improvements across bowel, bladder and prolapse‐related domains were greatest in the combined repair group. Health utility gains were observed in all cohorts. In patients with multicompartment prolapse, combined robotic repair was cost‐effective, whereas staged procedures were associated with higher cumulative costs without proportional gains in QALYs.

**Conclusions:**

Within robotic prolapse surgery, combined rectal and pelvic organ prolapse repair provides the most comprehensive symptom improvement and represents a cost‐effective strategy when both compartments are symptomatic. These findings support systematic evaluation for multicompartment prolapse and multidisciplinary surgical planning.

**What does this paper add to the literature?**

This study provides prospective multicentre data on functional outcomes and cost‐effectiveness of combined versus isolated robotic prolapse repair. It demonstrates that addressing both rectal and pelvic organ prolapse concurrently yields broader symptom improvement and avoids the higher cumulative costs associated with staged procedures.

## INTRODUCTION

Rectal prolapse (RP) and pelvic organ prolapse (POP) are complex pelvic floor disorders that frequently coexist in older women, leading to significant morbidity including obstructed defaecation, faecal incontinence, urinary symptoms and pelvic pressure [1, 2]. While RP is traditionally managed by colorectal surgeons and POP by urogynaecologists or urologists, the co‐occurrence of these conditions in up to one‐third of patients has led to increasing recognition of the need for multidisciplinary evaluation and management [3]. However, combined surgical repair is not routinely performed, and the clinical and economic implications of addressing one versus both compartments during a single operation remain incompletely understood.

From a patient perspective, combined repair may reduce exposure to multiple anaesthetics, hospitalizations and recovery periods. From a health system perspective, treating both compartments concurrently may reduce redundant operating room time and postoperative resource utilization compared with staged procedures. Despite these potential advantages, combined repair remains variably adopted due to differences in referral patterns, surgical expertise and uncertainty regarding outcomes and costs.

Cost‐effectiveness analyses provide a framework to evaluate value by integrating clinical outcomes with healthcare costs, commonly using quality‐adjusted life years (QALYs) to quantify changes in health‐related quality of life [4]. In pelvic floor surgery, QALYs allow quantification of symptom relief, restoration of function and improved well‐being after intervention. As healthcare systems shift towards value‐based models, CEAs are increasingly used to guide resource allocation and patient‐centred care. Quantifying the relative benefit of combined surgery may inform decision‐making within established multidisciplinary pelvic floor programs.

The objective of this study was to compare patient‐reported outcomes and cost‐effectiveness of three clinically relevant surgical strategies within robotic prolapse surgery: robotic rectal prolapse repair (rRP), robotic pelvic organ prolapse repair (rPOP) and combined robotic rectal and pelvic organ prolapse repair (rRP + rPOP), using prospective multicentre data with 12‐month follow‐up. This study was not designed to compare robotic surgery with alternative operative approaches, but rather to inform decision‐making regarding the management of single‐ versus multicompartment prolapse among patients selected for a robotic approach.

## MATERIALS AND METHODS

### Study population

This study was a prospective, multicentre cohort trial conducted at three tertiary academic centres in the United States with established multidisciplinary pelvic floor programmes: Stanford University, Massachusetts General Hospital and Corewell Health. Institutional Review Board (IRB) approval was obtained at all sites.

Between 2021 and 2023, women with full‐thickness rectal prolapse (FTRP), POP or both, who were scheduled for robotic surgery were screened and invited to participate. Surgical treatment consisted of robotic rectopexy (rRP), robotic pelvic organ prolapse surgery (rPOP) or combined robotic rectal and pelvic organ prolapse surgery (rRP + rPOP).

### Inclusion and exclusion criteria

Eligible participants were women aged 18 years or older with a diagnosis of FTRP and/or POP Quantification (POP‐Q) stage II–IV. All patients were required to speak English and to be capable of completing electronic surveys before and after surgery.

Exclusion criteria included pregnancy, slow‐transit constipation, planned perineal‐only rRP or transvaginal‐only POP surgery, or an untreated psychiatric illness that could affect the ability to complete follow‐up assessments. Patients were excluded if they were unable to complete surveys at baseline and follow‐up.

### Patient data

FTRP was diagnosed by visualization in the clinic or photograph. POP‐Q staging was performed on all patients. Demographic characteristics, preoperative comorbidities and surgical history were abstracted from the electronic medical record. The Charlson Comorbidity Index (CCI) was used to quantify comorbidity burden. Preoperative assessments, imaging, surgical decision‐making and operative technique were left to the discretion of the treating team and were confirmed with operative reports. Operative data included procedure type, mesh use, estimated blood loss, operating time and intraoperative complications that were recorded. Postoperative complications within 30 days were classified using the Clavien–Dindo system.

### Surgical procedures

Robotic rectal prolapse repair consisted of either ventral mesh rectopexy or suture rectopexy, performed according to surgeon preference and patient‐specific factors. Robotic pelvic organ prolapse repair included sacrocolpopexy or uterosacral ligament suspension, with or without concomitant vaginal repairs or hysterectomy. In combined procedures, rectal and pelvic organ prolapse repairs were performed during the same operative session in a coordinated, multidisciplinary fashion. The sequence of procedures and technical details were determined by the operating teams and documented in operative reports.

### Patient‐reported outcome measures

Validated, condition‐specific patient‐reported outcome measures (PROMs) were administered at baseline, 6 months and 12 months postoperatively using secure REDCap databases at each study site. These included: Pelvic Floor Distress Inventory (PFDI‐20) and Pelvic Floor Impact Questionnaire (PFIQ‐7) to evaluate prolapse, bowel and urinary symptom severity and impact on quality of life, Wexner and Vaizey scores to assess faecal incontinence, Faecal Incontinence Quality of Life (FIQoL) scale, Patient Assessment of Constipation Symptoms (PAC‐SYM) and Patient Assessment of Constipation Quality of Life (PAC‐QOL), Hospital Anxiety and Depression Scale (HADS) to evaluate psychosocial well‐being, POP /Incontinence Sexual Questionnaire IUGA‐Revised (PISQ‐IR) to evaluate sexual function, EuroQol‐5 Dimension 3 Level (EQ‐5D‐3L) for global health utility and Patient Global Impression of Change (PGIC). Both the Wexner and Vaizey scores were pre‐specified and retained because they capture complementary dimensions of faecal incontinence: the Wexner score weights episode frequency and pad use, while the Vaizey score adds urgency and the inability to defer defaecation. PROMs were completed electronically at each time point.

### Cost data and analysis

#### Economic evaluation design

A planned cost‐effectiveness analysis was conducted from the health care sector perspective using trial‐based data. The objective was to assess the incremental cost per QALY gained at 12 months, comparing three surgical strategies: robotic rectopexy only (rRP), robotic POP surgery only (rPOP) and combined robotic rectal and POP surgery (rRP + rPOP). Our approach followed current guidelines for cost‐effectiveness analysis. The analysis timeframe matched the duration of the trial (12 months). All costs were measured in 2024 US dollars. Discounting of costs or QALYs was not performed due to the one‐year analytic time horizon.

#### Effects

The primary effectiveness outcome for the cost‐effectiveness analysis was QALYs at 12 months. QALYs integrate survival and health‐related quality of life on a 0–1 utility scale. A gain of 1 QALY reflects 1 year of life in perfect health. QALYs were calculated from EQ‐5D‐3L responses collected at baseline, 6 months and 12 months. Individual responses were summarized into a single, preference‐weighted index score using US‐based value sets.

#### Costs

Direct medical costs from the institutional perspective were captured using billing and clinical cost data available at each study site. These included costs associated with operating room time, anaesthesia, surgical procedural reimbursement (based on CPT codes) and disposable surgical equipment. All costs of the index surgical episode were captured regardless of the specific combination of procedures performed, including any concomitant hysterectomy, salpingectomy, vaginal repair or anti‐incontinence procedure. We also collected cost data related to 30‐day postoperative complications (e.g. treatment for urinary tract infections or wound complications), emergency department visits, readmissions, reoperations and all in‐office follow‐up visits related to the index surgery within 12 months postoperatively.

Due to institutional restrictions, costs were aggregated by site and reported as relative values (incremental cost above rPOP), preserving internal validity for ICER estimation but limiting generalizability. We did not include preoperative costs (e.g. workup, imaging, anorectal manometry, urodynamics), and we excluded indirect costs such as lost productivity or transportation expenses. Only costs captured within the respective hospital systems were included in the analysis.

#### Cohorts

In our analysis, we evaluated the cost‐effectiveness of patients undergoing rRP surgery for RP alone and those undergoing rPOP surgery for POP alone. To better understand the subgroup of patients presenting with both rectal and POP, and to facilitate more informed economic decision‐making, we created a theoretical cohort divided into four treatment strategies: (1) patients with combined rectal and POP who undergo rRP only; (2) patients with combined prolapse who undergo rPOP only; (3) patients with combined prolapse who undergo a combined rRP + rPOP procedure; and (4) patients with combined prolapse who undergo staged procedures, with rectal and pelvic organ prolapse repairs performed in two separate surgeries. Staged procedures were included as a comparator to reflect real‐world clinical scenarios that cannot be ethically randomized. This cohort is a decision‐analytic construct rather than an empirical observation, and its results should be interpreted as directional rather than definitive given the small underlying sample.

### Statistical analysis

Descriptive statistics were used to summarize demographic, surgical and clinical characteristics across the three surgical groups (rRP, rPOP and rRP + rPOP). Continuous variables were compared using Kruskal–Wallis tests due to non‐parametric distribution, and categorical variables were compared using Fisher's exact tests.

Longitudinal outcomes, including changes in quality‐of‐life measures and functional scores, were analysed using multivariable linear mixed‐effects models. These models included fixed effects for time point (baseline, 6 months and 12 months), surgical group and the interaction between surgical group and time. Models were adjusted for patient age and site, with random intercepts for subjects to account for repeated measures. Within‐group change from baseline to 12 months for each group, with its 95% confidence interval and *p*‐value, was derived from these models.

For the cost‐effectiveness analysis, analysis of covariance (ANCOVA) was used to compare QALY gains across groups, adjusting for trial site and baseline EQ‐5D‐3L scores. Incremental cost‐effectiveness ratios (ICERs) were calculated using observed mean costs and QALYs for each group.

Sensitivity analyses determined whether changes in the model's input parameters altered the overall outcome of the cost‐effectiveness model. We conducted Tornado plots and multiple one‐way sensitivity analyses to determine if a threshold existed where the preferred strategy would change.

Cost modelling was performed using TreeAge Pro Healthcare 2023 and all statistical analyses were performed using SAS version 9.4 (SAS Institute, Cary, NC). Two‐sided *p*‐values <0.05 were considered statistically significant.

## RESULTS

Forty‐seven patients were included: 14 underwent rRP only, 16 underwent robotic pelvic organ prolapse repair only (rPOP) and 17 underwent combined robotic repair (rRP + rPOP). Several baseline characteristics differed significantly across groups, in a pattern consistent with surgical indication: history of depression (*p* = 0.03), prior RP surgery (*p* = 0.004), prior vaginal delivery (*p* = 0.008), POP‐Q stage (*p* < 0.001) and prior pelvic floor imaging (*p* = 0.004) (Table 1). All longitudinal models were adjusted for age and site to account for this baseline imbalance. Operating times were longest for combined procedures, and both combined and rRP groups had longer hospital stays. Complications were highest in the combined rRP + rPOP group, with two patients readmitted and one patient requiring a return to the operating room for a Clavien‐Dindo grade 3b complication (Table 2). In the combined group, the two Grade 1 complications were urinary retention, the Grade 2 complication was pneumonia with pulmonary embolism, and the Grade 3b complication was a pelvic abscess requiring return to the operating room. The single Grade 2 complication in the rRP group was a urinary tract infection.

**TABLE 1:** Demographics and baseline characteristics by treatment group.

Characteristic	rRP (*n* = 14)	rPOP (*n* = 16)	rRP + rPOP (*n* = 17)	Total (*n* = 47)	*p*‐value*
Age, median (IQR)	60.0 (41.0, 67.0)	66.5 (59.0, 70.0)	66.0 (63.0, 71.0)	65.0 (57.0, 71.0)	0.22**
BMI, median (IQR)	22.9 (19.3, 26.4)	24.9 (22.9, 28.8)	23.6 (22.5, 26.2)	24.5 (21.7, 27.8)	0.22**
Race					
White, non‐Hispanic	13 (93%)	16 (100%)	16 (94%)	45 (96%)	0.75
Other	1 (7%)	0	1 (6%)	2 (4%)	
CCI, median (IQR)	1 (0, 2)	0.5 (0, 2)	1 (1, 2)	1 (0, 2)	0.88**
History of depression	6 (43%)	1 (6%)	7 (41%)	14 (30%)	0.03
History of RP surgery	4 (29%)	0	8 (47%)	12 (26%)	0.004
History of POP surgery	1 (7%)	6 (38%)	4 (24%)	11 (23%)	0.17
History of hysterectomy	5 (36%)	11 (69%)	6 (35%)	22 (47%)	0.12
History of vaginal delivery	8 (57%)	16 (100%)	12 (71%)	36 (77%)	0.008
POP‐Q stage^a^					
0	4 (33%)	0	1 (6%)	5 (12%)	<0.001**
1	2 (17%)	0	0	2 (5%)	
2	6 (50%)	4 (29%)	12 (71%)	22 (51%)	
3	0	6 (43%)	4 (24%)	10 (23%)	
4	0	4 (29%)	0	4 (9%)	
Pelvic floor imaging	5 (36%)	0	8 (47%)	13 (28%)	0.004

^a^ POP‐Q stage available for 12 rRP, 14 rPOP and 17 rRP + rPOP patients; percentages calculated on patients with available staging.

* Fisher exact test for categorical variables unless otherwise noted.

** Kruskal–Wallis test for continuous variables (median, IQR).

**TABLE 2:** Operative details and 30‐day complications by treatment group.

Characteristic	Description	rRP (*n* = 14)	rPOP (*n* = 16)	rRP + rPOP (*n* = 17)	*p*‐value*
ASA^a^	I	0	1 (8%)	0	0.67
	II	10 (71%)	9 (69%)	11 (65%)	
	III	3 (21%)	3 (23%)	6 (35%)	
	IV	1 (7%)	0	0	
RP surgery type	Robotic ventral mesh rectopexy	9 (64%)		13 (76%)	0.69
	Robotic suture rectopexy	5 (36%)		4 (24%)	
Type of mesh for RP repair^b^	Synthetic	0		4 (33%)	0.10
	Biologic	9 (100%)		8 (67%)	
POP surgery type^c^	Robotic sacrocolpopexy		15 (94%)	8 (53%)	0.03
	Robotic uterosacral ligament suspension		1 (6%)	5 (33%)	
	Vaginal repair		0	2 (13%)	
POP concurrent procedures^d^	Hysterectomy		5 (31%)	5 (31%)	1.000
	Salpingectomy		7 (44%)	5 (31%)	0.72
	Oophorectomy		2 (13%)	2 (13%)	1.000
	Cystocele repair		7 (44%)	3 (19%)	0.25
	Perineorrhaphy		4 (25%)	1 (6%)	0.33
	Rectocele repair		7 (44%)	7 (44%)	1.000
	Urinary incontinence procedure		5 (31%)	5 (31%)	1.000
Total operating time (min), median (IQR)		181 (160, 210)	202 (170, 225)	278 (258, 305)	<0.001**
Length of stay (days), median (IQR)		1 (1, 1)	0 (0, 0)	1 (1, 2)	<0.001**
Clavien score	No complications	13 (93%)	16 (100%)	13 (76%)	0.38
	1	0	0	2 (12%)	
	2	1 (7%)	0	1 (6%)	
	3b	0	0	1 (6%)	
Readmission		0	0	2 (12%)	0.32

^a^ ASA class available for 13 of 16 rPOP patients; percentages calculated on available data.

^b^ Mesh type applies only to patients undergoing ventral mesh rectopexy (9 rRP, 13 rRP + rPOP); type available for 12 of 13 rRP + rPOP patients.

^c^ POP surgery type available for 15 of 17 rRP + rPOP patients.

^d^ Concurrent procedure data available for 16 of 17 rRP + rPOP patients.

* Fisher exact test for categorical variables unless otherwise noted.

** Kruskal–Wallis test for continuous variables (median, IQR).

At 12 months, all three cohorts demonstrated significant improvements in pelvic floor symptoms. The adjusted mean change in PFDI‐20 scores from baseline to 12 months was −47.3 for rRP, −81.1 for rPOP and −82.6 for rRP + rPOP (all within‐group *p* < 0.001; Table 3), with greater improvement in the rPOP and rRP + rPOP groups compared to rRP alone. As expected, prolapse‐specific domains (POPDI scores) improved most in the rPOP (−38.32) and rRP + rPOP group (−25.18), while colorectal‐specific domains (CRADI scores) improved most in the rRP (−31.57) and rRP + rPOP group (−26.83) (Table 3).

**TABLE 3:** Change from baseline to 12 months for bowel and bladder symptom scores by treatment group (rRP, rPOP and rRP + rPOP).

Measure	Cohort	Baseline, mean (SD)	12 months, mean (SD)	Mean change, baseline to 12 months (95% CI)^a^	Change *p*‐value^a^
PFDI‐20	rRP	100.04 (49.04)	42.50 (22.96)	**−47.34 (−72.38, −22.29)**	**0.0003**
	rPOP	105.41 (52.96)	22.37 (26.54)	**−81.12 (−101.20, −61.03)**	**<0.0001**
	rRP + rPOP	123.30 (55.12)	34.30 (30.28)	**−82.57 (−104.21, −60.92)**	**<0.0001**
	*p*‐value^b^	0.54	0.09		
POPDI	rRP	21.25 (19.08)	7.08 (6.82)	−9.54 (−19.28, 0.19)	0.0524
	rPOP	44.46 (20.64)	5.26 (8.29)	**−38.32 (−46.16, −30.48)**	**<0.0001**
	rRP + rPOP	33.65 (21.59)	6.55 (10.81)	**−25.18 (−33.60, −16.75)**	**<0.0001**
	*p*‐value^b^	0.02	0.65		
CRADI	rRP	57.37 (24.07)	23.75 (14.45)	**−31.57 (−42.95, −20.18)**	**<0.0001**
	rPOP	21.73 (20.21)	9.77 (11.63)	**−12.51 (−21.67, −3.36)**	**0.0080**
	rRP + rPOP	48.72 (15.63)	20.31 (20.17)	**−26.83 (−36.67, −16.98)**	**<0.0001**
	*p*‐value^b^	<0.001	0.05		
UDI	rRP	21.43 (15.92)	11.67 (9.58)	−6.82 (−19.77, 6.14)	0.2977
	rPOP	39.22 (30.41)	7.34 (10.80)	**−30.47 (−40.89, −20.04)**	**<0.0001**
	rRP + rPOP	40.94 (32.17)	7.44 (12.03)	**−31.25 (−42.45, −20.04)**	**<0.0001**
	*p*‐value^b^	0.18	0.19		
PFIQ‐7	rRP	80.63 (51.55)	28.00 (29.36)	**−51.44 (−87.56, −15.32)**	**0.0050**
	rPOP	75.30 (66.03)	10.22 (13.60)	**−65.15 (−96.18, −34.11)**	**<0.0001**
	rRP + rPOP	123.58 (81.06)	17.78 (22.00)	**−105.32 (−136.42, −74.21)**	**<0.0001**
	*p*‐value^b^	0.11	0.18		
POPIQ	rRP	7.62 (20.97)	0.95 (3.01)	−6.47 (−20.38, 7.43)	0.3494
	rPOP	26.19 (26.60)	2.08 (5.21)	**−24.20 (−36.16, −12.25)**	**<0.0001**
	rRP + rPOP	27.26 (34.73)	0 (0)	**−26.97 (−38.95, −14.99)**	**0.0001**
	*p*‐value^b^	0.05	0.22		
CRAIQ	rRP	54.92 (29.07)	19.43 (19.01)	**−34.33 (−49.52, −19.13)**	**<0.0001**
	rPOP	17.56 (27.48)	4.76 (7.58)	−12.92 (−25.91, 0.07)	0.0511
	rRP + rPOP	59.06 (22.40)	13.02 (16.54)	**−46.00 (−59.03, −32.98)**	**<0.0001**
	*p*‐value^b^	<0.001	0.11		
UIQ	rRP	18.10 (29.25)	7.62 (16.22)	−10.11 (−25.07, 4.85)	0.1792
	rPOP	31.55 (23.77)	3.37 (5.56)	**−28.06 (−40.80, −15.32)**	**<0.0001**
	rRP + rPOP	37.25 (33.68)	4.76 (9.35)	**−31.85 (−44.63, −19.08)**	**<0.0001**
	*p*‐value^b^	0.08	0.88		
PAC‐SYM	rRP	1.46 (0.91)	0.65 (0.45)	**−0.67 (−1.06, −0.27)**	**0.0013**
	rPOP	0.73 (0.79)	0.39 (0.33)	**−0.35 (−0.68, −0.02)**	**0.0373**
	rRP + rPOP	1.44 (0.64)	0.62 (0.52)	**−0.84 (−1.18, −0.50)**	**<0.0001**
	*p‐*value^b^	0.005	0.26		
Wexner	rRP	11.33 (5.95)	7.30 (6.50)	**−3.37 (−5.81, −0.94)**	**0.0074**
	rPOP	3.47 (4.42)	1.47 (2.26)	−1.83 (−3.87, 0.22)	0.0792
	rRP + rPOP	12.13 (4.57)	4.38 (4.74)	**−7.46 (−9.64, −5.28)**	**<0.0001**
	*p*‐value^b^	<0.001	0.02		
Vaizey	rRP	12.31 (6.66)	9.10 (5.45)	**−2.90 (−5.63, −0.17)**	**0.0378**
	rPOP	5.29 (6.09)	2.81 (2.83)	**−2.52 (−4.88, −0.15)**	**0.0373**
	rRP + rPOP	13.07 (4.33)	5.64 (4.68)	**−7.16 (−9.52, −4.81)**	**<0.0001**
	*p*‐value^b^	0.003	0.007		
PAC‐QOL	rRP	2.18 (0.89)	1.45 (0.61)	**−0.65 (−1.04, −0.27)**	**0.0012**
	rPOP	1.49 (0.80)	1.12 (0.50)	**−0.36 (−0.69, −0.04)**	**0.0296**
	rRP + rPOP	1.95 (0.62)	1.30 (0.58)	**−0.65 (−0.98, −0.33)**	**0.0002**
	*p*‐value^b^	0.02	0.22		
FIQoL	rRP	10.59 (3.18)	13.94 (1.74)	**2.82 (0.91, 4.72)**	**0.0046**
	rPOP	13.23 (3.48)	14.08 (1.93)	0.93 (−1.36, 3.21)	0.4197
	rRP + rPOP	10.11 (2.73)	13.45 (1.85)	**3.60 (2.02, 5.19)**	**<0.0001**
	*p‐*value^b^	0.21	0.72		

*Note*: Bold values indicate a statistically significant change from baseline (95% CI excludes zero).

^a^ Change from baseline to 12 months and corresponding *p*‐value estimated from linear mixed‐effects models, adjusted for age.

^b^ Kruskal–Wallis test for between‐group differences at each timepoint.

Quality‐of‐life improvements as measured by the PFIQ‐7 mirrored the PFDI‐20 trends. The adjusted mean PFIQ‐7 change was −51.4 for rRP, −65.2 for rPOP and −105.3 for rRP + rPOP (all within‐group changes statistically significant; Table 3). Adjusted mean changes in POPIQ scores were greatest for the rPOP and rRP + rPOP groups (−24.2 and −26.97, respectively), while CRAIQ scores improved most in the rRP and rRP + rPOP (−34.33 and −46, respectively).

Faecal incontinence severity, measured by Wexner and Vaizey scores, improved significantly in all groups, with the most pronounced changes in the rRP + rPOP group. Wexner scores declined by 7.5 points in rRP + rPOP patients (*p* < 0.01), compared to 3.4 points in the rRP and 1.8 points in the rPOP patients. Similar trends were observed in Vaizey scores (Table 3).

Constipation‐related quality of life, as assessed by PAC‐SYM and PAC‐QOL, improved across all groups, with the greatest improvements in the rRP and rRP + rPOP cohorts (Table 3).

Hospital Anxiety and Depression Scale (HADS) scores decreased most significantly in the rRP + rPOP group, with a −2.62‐point improvement in anxiety scores and a −2.02‐point improvement in depression scores (Table 4).

**TABLE 4:** Change from baseline to 12 months for quality‐of‐life scores by treatment group (rRP, rPOP and rRP + rPOP).

Measure	Cohort	Baseline, mean (SD)	12 months, mean (SD)	Mean change, baseline to 12 months (95% CI)^a^	Change *p*‐value^a^
HADS Anxiety	rRP	8.00 (5.75)	4.88 (3.72)	−1.81 (−4.04, 0.41)	0.1098
	rPOP	4.80 (3.57)	3.08 (2.64)	−1.47 (−3.36, 0.42)	0.1252
	rRP + rPOP	7.00 (3.44)	4.18 (3.03)	**−2.62 (−4.55, −0.70)**	**0.0092**
	*p*‐value^b^	0.15	0.47		
HADS Depression	rRP	4.29 (4.03)	1.63 (1.60)	−2.04 (−4.16, 0.07)	0.0595
	rPOP	2.36 (2.62)	1.83 (1.80)	−0.74 (−2.58, 1.11)	0.4246
	rRP + rPOP	4.63 (3.38)	2.73 (2.37)	**−2.02 (−3.87, −0.17)**	**0.0301**
	*p*‐value^b^	0.11	0.51		
PISQ‐IR	rRP	3.13 (0.71)	3.38 (0.58)	0.18 (−0.19, 0.55)	0.3336
	rPOP	2.68 (0.84)	3.58 (0.53)	**0.86 (0.56, 1.17)**	**<0.0001**
	rRP + rPOP	3.00 (0.84)	3.60 (0.38)	**0.64 (0.32, 0.97)**	**0.0001**
	*p*‐value^b^	0.25	0.76		
PGIC, significant improvement (5–7)^c^	rRP	—	8 (80%)	—	—
	rPOP	—	16 (100%)	—	—
	rRP + rPOP	—	14 (93%)	—	—
	*p*‐value^b^		0.17		
EQ‐5D‐3L	rRP	0.71 (0.15)	0.93 (0.07)	**0.21 (0.07, 0.34)**	**0.0034**
	rPOP	0.83 (0.15)	0.89 (0.10)	0.06 (−0.05, 0.17)	0.2941
	rRP + rPOP	0.62 (0.27)	0.76 (0.16)	**0.15 (0.04, 0.26)**	**0.0075**
	*p*‐value^b^	0.03	0.008		
EQ‐5D‐3L (VAS)	rRP	66.54 (13.26)	78.10 (12.09)	10.54 (−1.79, 22.86)	0.0860
	rPOP	74.80 (20.07)	83.56 (8.51)	8.84 (−1.34, 19.02)	0.0794
	rRP + rPOP	66.19 (16.68)	72.27 (18.68)	6.12 (−4.13, 16.38)	0.4053
	*p*‐value^b^	0.19	0.13		

*Note*: Bold values indicate a statistically significant change from baseline (95% CI excludes zero). EQ‐5D‐3L (VAS) is reported on a 0–100 scale; positive change indicates improvement.

^a^ Change from baseline to 12 months and corresponding *p*‐value estimated from linear mixed‐effects models, adjusted for age.

^b^ Kruskal–Wallis test for between‐group differences at each timepoint.

^c^ PGIC significant improvement available for 10 rRP, 16 rPOP and 15 rRP + rPOP patients; percentages calculated on available data.

Sexual function, measured by PISQ‐IR, improved significantly in the rPOP and rRP + rPOP groups. Mean improvements from baseline to 12 months were + 0.86 in the rPOP group and + 0.64 in the rRP + rPOP group. No significant change was observed in the rRP group (Table 4).

On the PGIC, 80% of rRP patients, 100% of rPOP patients and 93% of rRP + rPOP patients reported meaningful symptom improvement at 12 months. Health utility, as measured by the EQ‐5D‐3L, showed QALY improvements of 0.21 for rRP, 0.06 for rPOP and 0.15 for rRP + rPOP at 12 months (Table 4). Between‐group comparisons should be interpreted cautiously given the limited sample size; however, improvements were consistently observed across multiple validated outcome measures.

From a cost perspective, rPOP was the least costly strategy, followed by rRP (an additional $10,090 per surgery), and then combined rRP + rPOP (an additional $16,693). Both rRP and rPOP were cost‐effective strategies, remaining below the willingness‐to‐pay threshold of $100,000 per QALY. The ICER for rRP compared to rPOP was $67,267 (Table 5).

**TABLE 5:** One‐year incremental cost, incremental effectiveness and incremental cost‐effectiveness ratio two prolapse models ranked by cost.

Model	Strategy	Incremental cost (2024 $US)	Incremental effectiveness (QALY)	ICER (2024 $US/QALY)
Patients with RP or POP	Robotic pelvic organ prolapse surgery (rPOP)	—	—	—
	Robotic rectal prolapse surgery (rRP)	$10,090.00	0.15	$67,266.67
Patients with both RP and POP	Robotic pelvic organ prolapse surgery (rPOP)	—		
	Robotic rectal prolapse surgery (rRP)	$10,090.00	0.18	$56,055.56
	Combined rectal prolapse and pelvic organ prolapse robotic surgery (rRP + rPOP)	$6,603.00	0.06	$83,333.33
	Staged rectal prolapse and pelvic organ prolapse robotic surgery (rRP or rPOP → rRP or rPOP)	$907.00	0	Dominated strategy

*Note*: First model: Patients with either POP or RP undergoing 1) robotic rectal prolapse repair (rRP) and 2) robotic pelvic organ prolapse repair (rPOP). Second model: Patients with both RP and POP undergoing 1) robotic rectal prolapse repair (rRP) only, 2) robotic pelvic organ prolapse repair (rPOP) only, 3) combined rectal prolapse and pelvic organ prolapse robotic repair (rRP + rPOP), 4) Staged rectal prolapse and pelvic organ prolapse robotic repair (rRP or rPOP ➔ rRP or rPOP).

In the theoretical cohort of patients with multicompartment prolapse, rRP, rPOP and combined rRP + rPOP surgery were all cost‐effective strategies remaining below the $100,000 per QALY threshold. The ICER was $56,056 for rRP and $83,333 for combined rRP + rPOP (Table 5). In contrast, performing two staged procedures—rRP or rPOP followed by rPOP or rRP was a dominated strategy and not cost‐effective.

**FIGURE 1:**
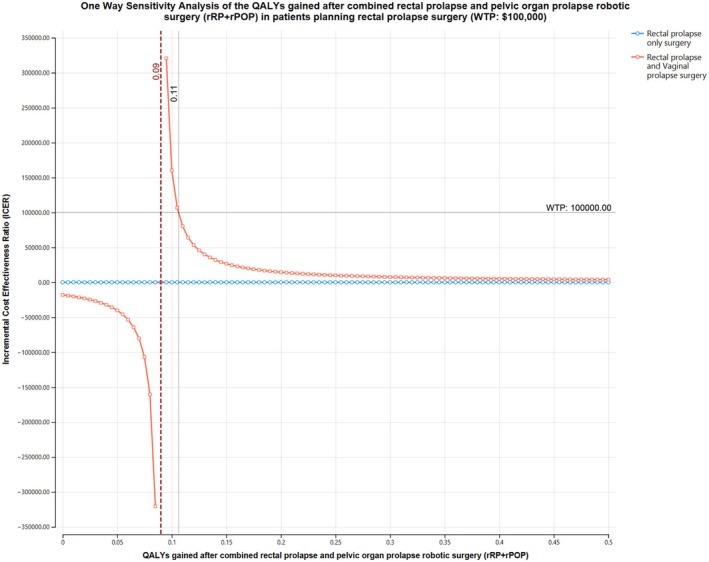
One‐way sensitivity analysis evaluating the impact of variation in quality‐adjusted life year (QALY) gain on the preferred surgical strategy in a theoretical cohort of patients with both rectal and pelvic organ prolapse who would otherwise undergo isolated robotic rectal prolapse repair. The analysis demonstrates the threshold at which combined robotic rectal and pelvic organ prolapse repair becomes the preferred cost‐effective strategy relative to isolated rectal prolapse repair, assuming a willingness‐to‐pay threshold of $100,000 per QALY. Vertical bars represent the range of plausible QALY values tested in the model.

**FIGURE 2:**
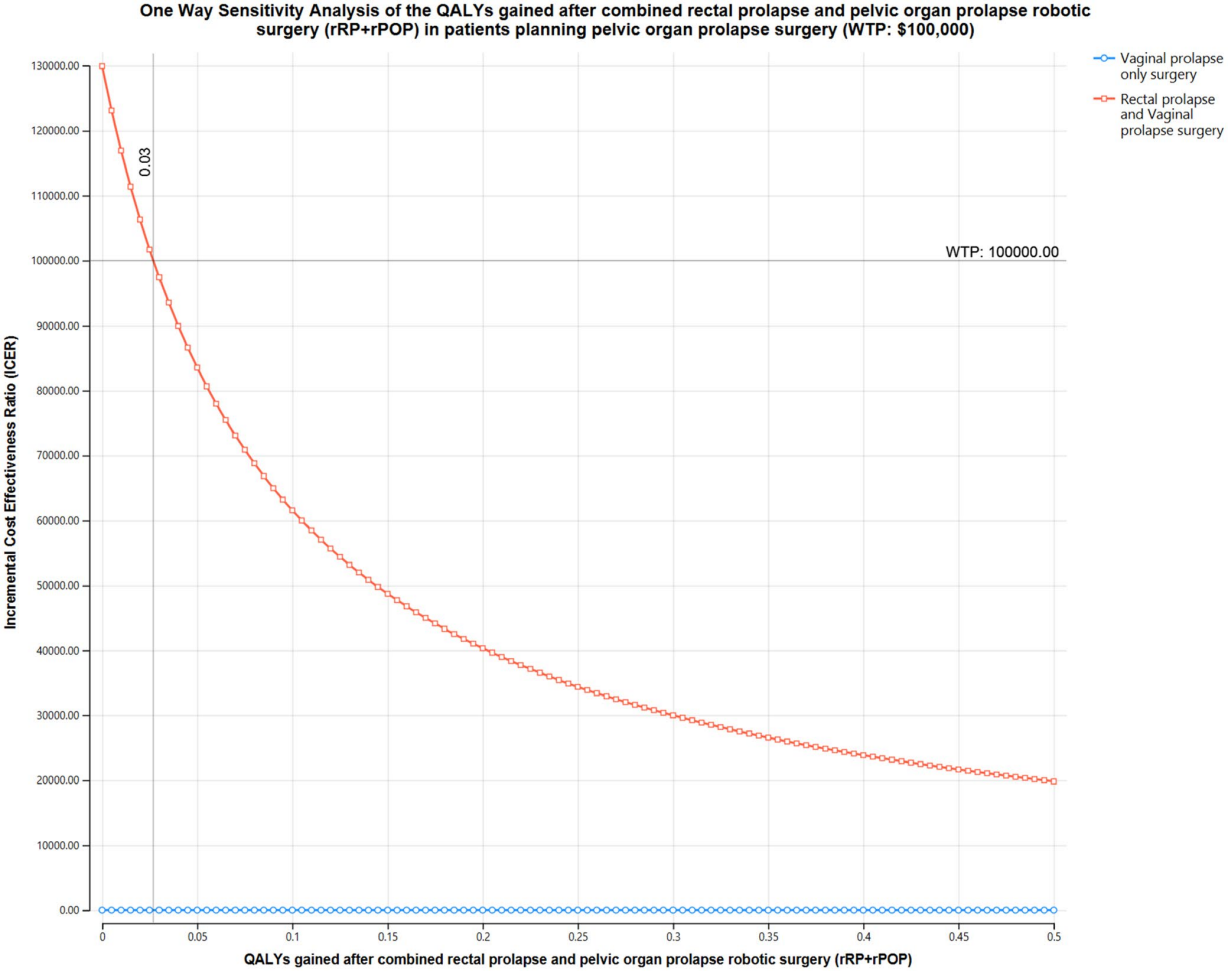
One‐way sensitivity analysis assessing the effect of variation in quality‐adjusted life year (QALY) gain on cost‐effectiveness in a theoretical cohort of patients with combined rectal and pelvic organ prolapse who would otherwise undergo isolated robotic pelvic organ prolapse repair. The figure illustrates the QALY threshold at which combined robotic repair becomes the preferred strategy under a willingness‐to‐pay threshold of $100,000 per QALY. This analysis highlights the sensitivity of cost‐effectiveness conclusions to changes in health utility estimates when both compartments are addressed concurrently.

In a one‐way sensitivity analysis, combined surgery became the most cost‐effective option if the QALY gain from rRP + rPOP exceeded 0.11 in patients who would otherwise undergo rRP alone for combined prolapse without treatment for POP (Figure 1). Similarly, combined surgery became the optimal strategy if the QALY gain exceeded 0.03 in patients who would otherwise undergo rPOP alone for combined prolapse without treatment for RP (Figure 2).

## DISCUSSION

In this prospective multicentre cohort study, we evaluated patient‐reported outcomes, health‐related quality of life and cost‐effectiveness of three clinically relevant robotic prolapse surgery strategies: isolated rRP, isolated pelvic organ prolapse repair and combined repair of both compartments. All three approaches were associated with statistically significant and clinically meaningful improvements in pelvic floor symptoms and quality of life at 12 months (Tables 3 and 4). However, the breadth and magnitude of symptom improvement were greatest among patients undergoing combined repair, particularly across bowel, bladder and prolapse‐related domains.

From a cost‐effectiveness perspective, in our theoretical cohort of patients with symptomatic multicompartment prolapse, both isolated rRP and isolated pelvic organ prolapse repair represented cost‐effective strategies within accepted willingness‐to‐pay thresholds. However, patients in these two cohorts were modelled as retaining untreated symptoms from the other compartment, leading to smaller improvements in QALYs. In this theoretical cohort, combined robotic repair was also cost‐effective, remaining below the willingness‐to‐pay threshold of $100,000 per QALY. Although combined procedures incurred higher upfront institutional costs, these were offset by broader symptom improvement and by avoidance of cumulative costs associated with staged operations.

In contrast, staged repair strategies were economically unfavourable due to the additional costs of a second operation without proportional incremental gains in health utility. These findings emphasize that combined repair provides the most efficient and clinically beneficial approach for appropriately selected patients with dual‐compartment prolapse.

The observed QALY gains in this study were consistent with those reported in other studies of pelvic reconstructive surgery [5]. The isolated rRP cohort showed the highest QALY improvement (+0.21), followed by combined repair (+0.15) and isolated pelvic organ prolapse repair (+0.06) [6, 7, 8]. These gains corresponded with functional improvements in patient‐reported outcomes across PFDI, PFIQ, Wexner, Vaizey and PAC‐QOL scores [9, 10]. Of note, the combined repair cohort experienced the greatest improvement in composite symptom burden and quality‐of‐life scores, which may reflect the additive benefit of treating both compartments simultaneously in appropriately selected patients. Sexual function and mental health scores also improved most in the isolated rRP and combined repair cohorts, consistent with prior literature demonstrating improvements in these domains following prolapse repair [11, 12, 13]. PGIC scores at 12 months were highest in the isolated pelvic organ prolapse repair group but remained favourable in the combined group, suggesting high patient satisfaction despite the more extensive procedure.

Our study highlights that the EQ‐5D‐3L, while commonly used in cost‐effectiveness research, may not fully reflect pelvic floor–specific quality‐of‐life improvements. Although we observed greater improvement in both bladder and bowel symptoms among combined repair patients based on condition‐specific measures (e.g. PFDI, PFIQ, Wexner, Vaizey), these gains were only partially reflected in the QALY calculations derived from EQ‐5D‐3L. An additional limitation is that QALYs for the theoretical cohort were extrapolated from patients undergoing isolated rRP or isolated pelvic organ prolapse repair without multicompartment prolapse. It is possible that patients with combined prolapse who undergo isolated repair of only one compartment may experience different QALY gains, which could impact the cost‐effectiveness comparison.

A major strength of our study is the use of a theoretical cohort which allowed evaluation of clinically relevant scenarios that cannot be readily studied through randomized assignment. Patients with documented multicompartment prolapse may undergo incomplete surgical treatment of one compartment due to symptom prioritization or referral patterns. Modelling this scenario demonstrated that while symptom improvement may occur, the cumulative costs of staged treatment render this approach less efficient than concurrent repair when both compartments are symptomatic.

Additional strengths include the prospective design, multicentre collaboration, and consistent directionality of findings across multiple validated instruments which strengthen the relevance of the results. Importantly, this study was not designed to compare robotic surgery with alternative operative platforms and should be interpreted within the context of established robotic pelvic floor programs.

Our study has several limitations including modest sample size and the use of relative rather than absolute cost data. Recurrence of rectal or POP was not a pre‐specified outcome and is not captured within the 12‐month horizon; recurrence is a known driver of long‐term cost‐effectiveness in prolapse surgery, and longer follow‐up will be required to determine whether recurrence differs between strategies and alters the economic comparison. Surgical technique also varied across and within centres in the absence of a procedural consensus, which limits direct procedural comparison and the generalizability of cost estimates. We did not categorize the specific anatomical subtype of POP, that is apical or vault, uterine, anterior or posterior, as a discrete study variable, although subtype may influence recurrence and downstream costs. The cost analysis captured complication‐related costs within 30 days but did not capture complications beyond 30 days, indirect costs or recurrence‐ and reoperation‐related costs beyond the study window, all of which could affect longer‐term cost‐effectiveness. This study was also conducted at centres with integrated multidisciplinary pelvic floor clinics, where patients were routinely evaluated for both rectal and vaginal prolapse. In real‐world practice, coordinating dual evaluation and surgical planning may require multiple visits and interdepartmental scheduling, which incurs costs not captured in our 12‐month postoperative economic analysis.

The cost associated with the combined surgery group may have been disproportionately elevated due to the two readmissions and one return to the operating room in that group. Given the small sample sizes, these outlier events may have substantially influenced the overall cost estimates for the combined procedure. Several studies have shown that combined surgery is safe and complications are rare [14, 15]. It is possible that the combined surgery may be even more cost‐effective in a larger sample with more stable estimates of complication rates.

In patients undergoing robotic prolapse surgery, isolated rRP and isolated pelvic organ prolapse repair are both associated with meaningful improvements in symptoms and quality of life and represent cost‐effective treatment strategies. For patients with symptomatic multicompartment prolapse, combined robotic rectal and pelvic organ prolapse repair provides the most comprehensive improvement across bowel, bladder and prolapse‐related domains and is a cost‐effective approach when both compartments are addressed concurrently. In contrast, staged procedures were associated with higher cumulative costs without proportional gains in health utility. These findings support systematic evaluation for multicompartment prolapse and multidisciplinary, symptom‐driven surgical planning within established pelvic floor programs.

## AUTHOR CONTRIBUTIONS

**Michelle Earley:** Conceptualization; investigation; data curation; formal analysis; software; project administration; supervision; resources; methodology; validation; visualization; writing – original draft; writing – review and editing. **Raisa Platte:** Conceptualization; investigation; supervision; resources; writing – review and editing; writing – original draft; data curation. **Kavita Mishra:** Conceptualization; investigation; funding acquisition; writing – original draft; writing – review and editing; data curation; supervision; resources. **Liliana Bordeianou:** Conceptualization; investigation; funding acquisition; writing – original draft; writing – review and editing; supervision; formal analysis; project administration; resources. **Shannon L. Wallace:** Conceptualization; investigation; funding acquisition; writing – original draft; methodology; validation; visualization; writing – review and editing; data curation; supervision; resources; project administration. **James W. Ogilvie Jr.:** Conceptualization; investigation; writing – review and editing; formal analysis; project administration; supervision; resources; funding acquisition; writing – original draft. **Ekene A. Enemchukwu:** Conceptualization; investigation; funding acquisition; writing – original draft; writing – review and editing; data curation; supervision; resources. **Eric R. Sokol:** Conceptualization; investigation; funding acquisition; writing – original draft; writing – review and editing; data curation; supervision; resources. **Milena M. Weinstein:** Data curation; supervision; resources; writing – review and editing; writing – original draft; conceptualization; investigation; funding acquisition. **Brooke H. Gurland:** Conceptualization; investigation; funding acquisition; writing – original draft; methodology; validation; visualization; writing – review and editing; project administration; supervision; resources; data curation.

## FUNDING INFORMATION

American Society of Colon and Rectal Surgeons (ASCRS) Research Foundation (Research in Robotic Surgical Technology Grant).

## CONFLICT OF INTEREST STATEMENT

The authors declare no conflicts of interest except for the following: Speaker's Bureau: Ogilvie (Cook Medical), Gurland (Intuitive). Royalties: Bordeianou (UpToDate), Weinstein (UpToDate). Consultant: Bordeianou (CookMyosite), Weinstein (Materna Medica).

## ETHICS STATEMENT

This study was approved by the Institutional Review Board at each participating site. Stanford University served as the coordinating center (IRB number 46691). All participants provided written informed consent prior to enrollment. The study was conducted in accordance with the Declaration of Helsinki.

## Data Availability

The data that support the findings of this study are available on request from the corresponding author. The data are not publicly available due to privacy or ethical restrictions.
